# A Novel Arthropod Host of Brucellosis in the Arid Steppe Ecosystem

**DOI:** 10.3389/fvets.2020.566253

**Published:** 2020-10-23

**Authors:** Tianpeng Huang, Jinbao Zhang, Changyun Sun, Zhicheng Liu, Haiyan He, Jie Wu, Geriletu

**Affiliations:** ^1^College of Veterinary Medicine, Inner Mongolia Agricultural University, Hohhot, China; ^2^Department of Animal Husbandry and Veterinary Medicine, Zhalantun Vocational College, Hulun Buir, China; ^3^Guangdong Provincial Center for Disease Control and Prevention, Guangzhou, China

**Keywords:** *brucella*, *D nuttalli*, developmental stages, salivary gland, midgut, biotype

## Abstract

Brucellosis is a severe public health problem in the Inner Mongolia regions of China. The recent prevalence of brucellosis outbreaks may be attributed to an increase in the activity of ticks and other air-borne vectors. *Dermacentor nuttalli* (*D. nuttalli*) is a native tick species of Inner Mongolia; similar to other tick species, *D. nuttalli* carries a variety of pathogens that can be transmitted to a wide range of animals. In this study, we have investigated the potential of *D. nuttalli* in transmitting brucellosis. From 2015 to 2019, 2,256 ticks were collected from 23 different pastoral areas of Hulun Buir. Brucellosis pathogen was detected using DNA extracted from different developmental stages of ticks. Salivary gland and midgut tissue samples were used as templates to amplify *Brucella Bscp31* gene (*Brucella* genus-specific gene) by using TaqMan Real-Time polymerase chain reaction (PCR). To detect the presence of Bscp31 protein, which is specific to *Brucella* spp., in the midgut and salivary gland tissues of *D. nuttalli*, Western blotting and immunofluorescence were performed. Additionally, *Brucella* spp. were isolated using a culture medium. Tick samples were identified as *D. nuttalli*. Different percentages of *Brucella* genus-specific genes could be found in the tick samples. From 2015 to 2019, the positivity rate for the detection of *Bscp31* gene in *D. nuttalli* ranged from 0.00 to 87.80%, with the highest rate of 89.00%. In addition, *Brucella* genus-specific genes were successfully detected in the samples isolated from all the developmental stages and anatomical regions of ticks. Bscp31 protein was present in the midgut and salivary gland of *D. nuttalli*. Further, *B. melitensis* biotype 3 was isolated from eggs and engorged adults of *D. nuttalli*. These findings demonstrate that *D. nuttalli* is a potent, long-term carrier of *Brucella* spp. that can exhibit transovarial transmission potential, presenting *D. nuttalli* as a novel arthropod host for *Brucella* spp. This study, therefore, indicates the potential risk of transmission of brucellosis via tick bites among animals as well as human beings.

## Background

Brucellosis, a zoonotic infectious disease caused by *Brucella* spp., was once distributed worldwide (1); China effectively curbed it using attenuated vaccines in the 1980s (2, 3). However, in the last 10 years, China has seen a resurgence in the occurrence of brucellosis in animals. Alarmingly, there have also been reports of outbreaks of human brucellosis especially in Inner Mongolia (4). This disease poses serious problems against the healthy development of animal husbandry-associated economy and leads to the development of issues of social and public security (5, 6). Inner Mongolia has arid and semi-arid grassland type climate, but the global climate changes have impacted the inherent climatic characteristics of these areas (7). These climatic changes may have led to an alteration in vector-pathogen interactions and in turn created favorable conditions for the prevalence of certain infectious diseases (8). The genus of *Brucella* can be divided into six species according to their biotypes, namely, *B. abortus, B. melitensis, B. ovis, B. canis, B. suis*, and *B. neotomae*. *B. melitensis* type 3 has emerged as the main epidemic-causing strain in Inner Mongolia (9, 10). Transmission of brucellosis in animals occurs mainly through the digestive tract, skin and mucous membranes, reproductive tract, and respiratory tract (11). However, it is worth noting that a rapid increase in the numbers of some common arthropods (such as mosquitoes, ticks, and flies) in the grasslands has occurred with the change in climate, which may be a probable reason behind the increase in the incidence of insect-transmitted diseases in these areas (12).

*Dermacentor nuttalli* is mainly a blood-sucking ectoparasite of animals that can also affect humans (13). Apart from causing inflammatory reactions such as emaciation, pain, and itch owing to direct blood sucking from an animal's body surface, *D. nuttalli* can transmit various infectious diseases caused by various pathogens (14–17). Therefore, the interspecific transmission, persistence, and increasing epidemic trend of brucellosis may be necessarily related to the activity of local arthropods. As early as 1944, Galuzo et al. have reported that ticks are among the reservoir vectors for brucellosis (18). Recently in 2017, Hosseini confirmed that *Boophilus* plays an intermediary role in the transmission of brucellosis (19). Wang et al. also reported that *Brucella* spp. responsible for bovine and ovine brucellosis were isolated from the eggs of *Dermacentor marginatus* collected in Xinjiang (20). However, these experimental data are too limited to confirm that ticks can carry *Brucella* spp. for a long time. In this study, from 2015 to 2019, a total of 2,256 ticks of the species *D. nuttalli* (including blood-sucking adult ticks and wild nymphal ticks) were collected from the Hulun Buir region. Using different methods, genes and proteins of *Brucella* spp. were detected in different sexes, developmental stages, and tissues of these ticks. More importantly, four strains of *B. melitensis* type 3 were successfully isolated from adult ticks and their eggs, which strongly proves that *D. nuttalli* might be one of the natural reservoir vectors that could carry *Brucella* spp. for a long time. It also suggested that *Brucella* spp. could remain epidemic among humans and animals for several years, which might be closely associated with the activity of *D. nuttalli* in this area.

## Materials and Methods

### Collection of Tick Samples

**Figure 1:**
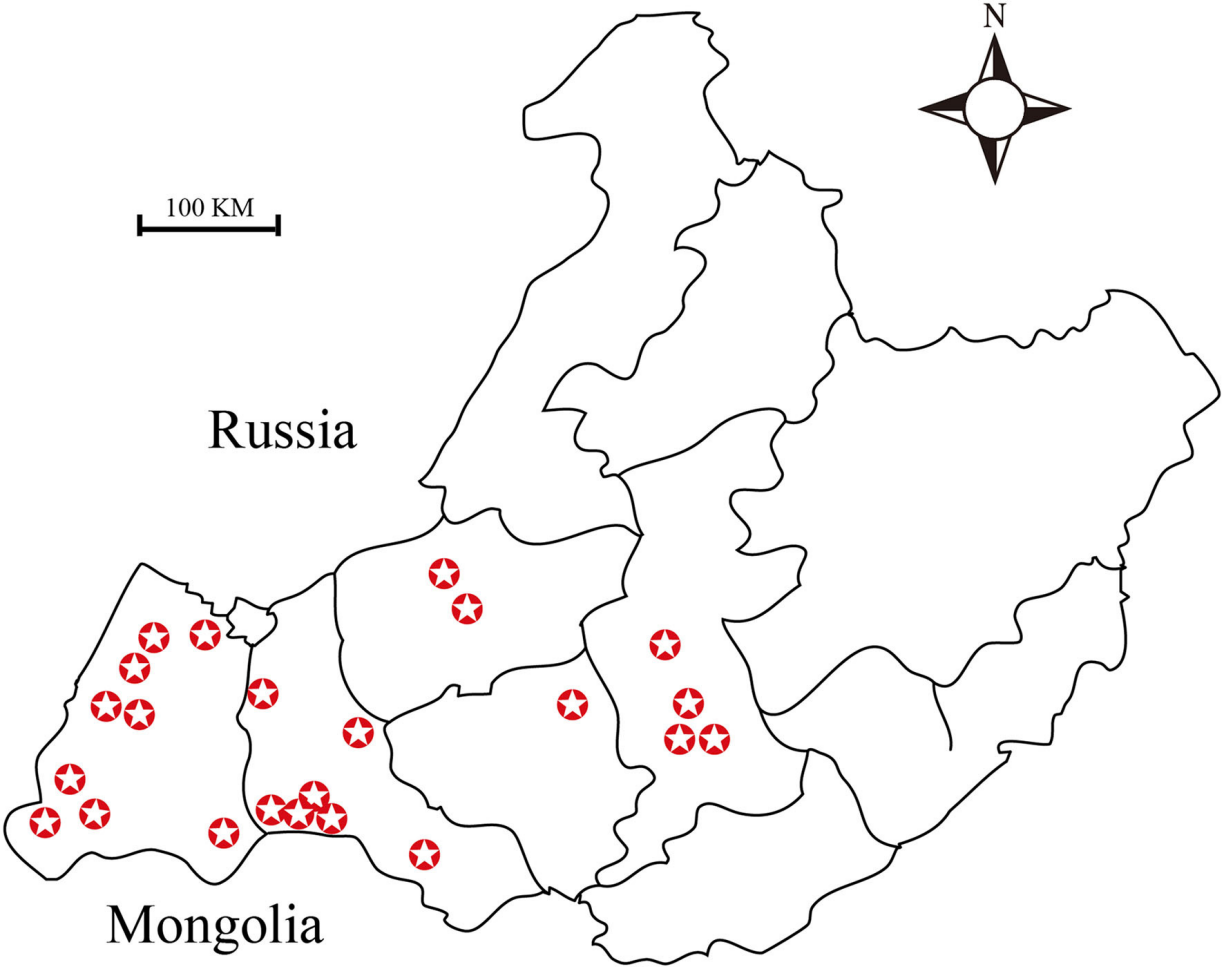
Map showing the sites from which ticks were collected. A total of twenty-three sampling sites have been highlighted in this map.

A total of 2,256 ticks were collected from 2015 to 2019, from Hulun Buir (115°31′ to 126°04′E, 47°05′ to 53°20′N) region of China either by dragging a sheet through an area with vegetation or directly from sheep (1) (Figure 1). Out of these, 1,911 ticks were used for DNA extraction. In addition, 50 engorged ticks were activated by artificial incubation in the laboratory. Moreover, 120 nymphal ticks were collected from the meadow, and 245 ticks were used for *Brucella* isolation test.

### Genomic DNA Extraction From the Ticks

For DNA extraction, 1,911 ticks were selected (21). The ticks were disinfected by washing with 70% ethanol for 10 min and then rinsed with PBS (pH 7.4). Each tick was grated into small pieces using sterile surgical blades. The grated samples were suspended in PBS and centrifuged. The first supernatant was discarded, and the pellet was then digested using lysis buffer (20 mM Tris-HCl pH 8.0, 1 mM EDTA pH 7.5, 10 mM NaCl, 1% SDS, and 100 μg/mL Proteinase K). DNA was extracted using the phenol-chloroform method. Following ethanol precipitation, the DNA samples were dissolved in 50 μL of double distilled water and then stored at −20°C until further use.

### Detection of *Brucella* Genus-Specific Genes in the Tick gDNA by Polymerase Chain Reaction (PCR)

#### Detection of *Brucella* Genus-Specific Genes From Tick gDNA

We used the previously reported *Brucella* genus-specific primers (22). The forward and reverse primer sequences used were 5′-TGGCTCGGTTGCCAATATCAA-3′ and 5′-CGCGCTTGCCTTTCAGGTCTG-3′, respectively. PCR amplification was carried out using the following parameters: initial denaturation at 95°C for 5 min; 30 cycles of 94°C for 30 s, 58°C for 30 s, 72°C for 1 min; and final extension at 72°C for 7 min. DNA samples from *Brucella melitensis* were used as a positive control.

#### PCR-Assisted Detection of *Brucella* Genus-Specific Genes From Ticks at Different Developmental Stages

Engorged female ticks were first disinfected using 70% alcohol and then transferred into a large sterilized tube plugged with cotton wool. The ticks were incubated at 25°C and 50% relative humidity. After spawning, some of the eggs were collected and cleaned; the remaining eggs were left to hatch. Some of the larvae were also collected and cleaned. Nymphs were also collected from meadows using a white cloth trap. Finally, all the samples were suspended in PBS and subsequently subjected to brief centrifugation. After centrifugation, the supernatant was discarded, and the pellet was used for gDNA isolation using the procedure described previously. The positive control used for this assay is same as that described in section Detection of Brucella Genus-Specific Genes From Tick gDNA.

### Quantification of *Brucella* Genus-Specific Genes Using TaqMan Real-Time PCR

In this analysis, the required primers were designed using the quantitative PCR tool of Integrated DNA Technologies (http://sg.idtdna.com). The forward and reverse primer sequences used were 5′-TCAATGCGATCAAGTCGG-3′ and 5′-GCATCCTTACGCGCAA-3′, respectively. The primer for the hybridization probe was FAM-5′-ATTGGGCCTATAACGGCACC-3′-TAMRA. We isolated the salivary glands and midguts from ticks under dissecting microscope (OLYMPUS BX51) and extracted DNA from them separately. These were used as template DNA. The reaction mixture (20 μL final volume) contained 10 pM of each primer, 4 pM of each hybridization probe, 10 μL of Premix Ex Taq, and 4 μL of DNA extract. PCR was performed using Applied Biosystem 7,500 and the following parameters: denaturation at 95°C for 2 min; 45 cycles of denaturation at 95°C for 15 s, and primer annealing at 58.5°C for 30 s. The experimental data were analyzed using GraphPad Prism 6 software.

### Detection of Bscp31 Protein of *Brucella* in *D. nuttalli*

#### Detection of Bscp31 Protein of *Brucella* in *D. nuttalli* by Immunofluorescence

Tick guts and salivary gland from engorged *D. nuttalli* were dissected in ice-cold PBS. All tissues were washed in PBS. To prepare samples for indirect fluorescence microscopy, dissected tissues were first placed in small plastic cassettes. After cooling, tissues were then sectioned using a SM2010R sliding microtome. Tissue sections (thickness = 25–30 μm) were placed on a glass slide, allowed to dry and then subjected to deparaffinization and dewaxing in xylene followed by hydration in successively decreasing concentrations of ethanol. Tissues were then permeabilized with 0.5% Triton X-100 in PBS for 30 min and washed three times with excessive quantities of PBS before blocking overnight with 5% BSA at 4°C. After washing again with PBS, the slides were then incubated for 1 h at 37°C followed by overnight incubation with polyclonal mouse anti-GST-Bscp31 antibodies (prepared in the laboratory). After washing the slides three times with PBS, secondary Alexa Fluor 488 antibodies (goat anti-mouse IgG conjugated with Alexa Fluor 488; Invitrogen) diluted to 1:100 in blocking solution were added to them. Secondary antibody incubation was carried out for 1 h at 37°C, in the dark. After a final PBS wash, slides were placed in a moist dark box to prevent the drying and fading of the fluorescence signal due to light exposure. Tick tissue sections were visualized under a Nikon C2 confocal microscope.

#### Detection of Bscp31 Protein of *Brucella* in *D. nuttalli* by Western Blot

Tick guts and salivary glands were dissected in cold PBS and lysed with RIPA buffer supplemented with 1 mM PMSF. The supernatant was collected after centrifugation at room temperature (25°C), at 14,000 × g for 10 min. The supernatants obtained were used as the samples for Western blotting. Samples were electrophoresed for 1 h 30 min at 100 V constant current. The SDS-PAGE gel was transferred to a PVDF membrane for 1 h at 100 V. The membrane was blocked with 10% skimmed milk in TBST for 5 h at room temperature (25°C), followed by three washes with TBST. It was then incubated overnight with purified anti-GST-Bscp31 antibody (prepared in the laboratory) diluted to 1:500 in TBST. Following this, the membrane was washed three times with TBST and incubated in secondary antibodies (HRP-conjugated goat anti-mouse; Abcam; 1:2000 dilution) for 2 h, in the dark at room temperature (25°C). Protein bands in the membrane were visualized after staining with enhanced chemiluminescence solution.

### Isolation and Identification of *Brucella* spp. in *D. nuttalli*

#### Culture and Phenotypic Identification of *Brucella* spp. in *D. nuttalli*

The washed engorged female ticks and eggs were crushed using sterilized micro pestles. Lysates were then suspended in 60 μL sterilized saline water. The mixtures were streaked onto *Brucella* selective media (Hopebio, China) plates and cultured in an incubator at 37°C for 72 h. During this period, different bacterial colonies were transferred to blood agar plate (identified according to their sizes and morphological features) and subsequently re-cultured. The suspected *Brucella* colonies were identified by Gram staining and PCR-based detection of *Bscp31* gene. AMOS-PCR was used to identify *Brucella* species (23), and this was followed by characterization using the classical *Brucella* phenotypic identification procedures, such as CO_2_ requirement, H_2_S production, dye sensitivity (toasic fuchsin and thionin), agglutination with mono-specific antisera, and phage typing. The phenotypic identification tests were run in triplicates. The *Brucella* isolation test was carried out in the BSL-3 laboratory.

### Phylogenetic Tree Analyses of 16S rRNA Genes of *Brucella*

The 16S rRNA gene were amplified using the methods previously described by Scholz et al. The forward and reverse primers 27F (5′-AGAGTTTGATCCTGGCTCAG-3′) and 1492R (5′-GGTTACCTTGTTACGACTT-3′) were used to generate an ~1,400 bp long fragment of the 16S rRNA gene. The parameters of PCR cycle were as follows: 94°C for 5 min, 34 cycles at 94°C for 60 s, 56°C for 30 s, 72°C for 90 s, and 10 min at 72°C. The PCR products were analyzed by gel electrophoresis in 1% agarose. The PCR products were sent to TIANYI HUIYUAN for sequencing. Phylogenetic analyses were conducted by comparing reference sequences from GenBank with the four sequences obtained from this study, using the MEGA 6 software (24). The neighbor-joining method was employed to construct a phylogenetic tree. The reliability of the tree was assessed using bootstrap analysis with 1,000 replicates.

## Results

### Detection of *Brucella* Genus-Specific Genes by PCR

#### Detection of *Brucella* Genus-Specific Genes in Tick Samples

Details of the *Brucella* genus-specific genes detected in ticks collected during 2015–2019 are presented in Supplementary Table 1. The positivity rate ranged from 0.00 to 87.80% in different sampling regions. Interestingly, the positivity rate was 0.00–81.82% in female ticks and 0.00–92.41% in male ticks (Supplementary Table 1).

#### Detection of *Brucella* Genus-Specific Genes at Different Developmental Stages

**Figure 2:**
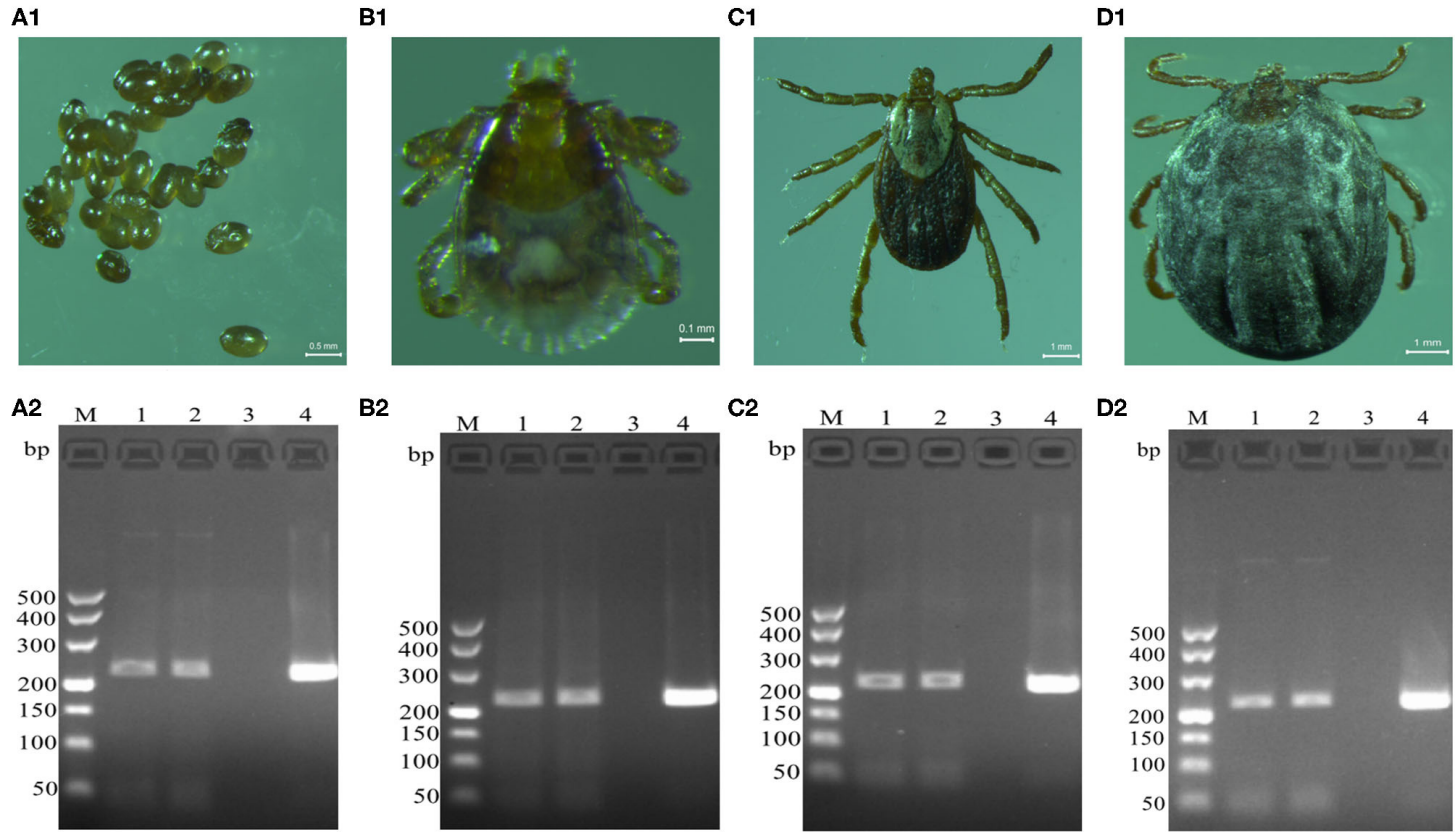
Detection of *Brucella* genus-specific *Bscp31* gene in different developmental stages of *D. nuttalli. Brucella* genus-specific genes were detected in different developmental stages. **(A1)** Eggs. **(B1)** Larva. **(C1)** Nymph. **(D1)** Adult female. M: DNA Markers (A): 500, 400, 300, 200, and 100 bp, respectively. **(A2)** Detection of *Bscp31* gene in eggs of *D. nuttalli*. **(B2)** Detection of *Bscp31* gene in larvae of *D. nuttalli*. **(C2)** Detection of *Bscp31* gene in nymphs of *D. nuttalli*. **(D2)** Detection of *Bscp31* gene in adults of *D. nuttalli*. Lanes 1-2: DNA samples from *D. nuttalli*. Lane 3: Negative control. Lane 4: Positive control (DNA samples from *Brucella melitensis*).

*Brucella* genus-specific genes were detected in different developmental stages (eggs, larva, nymph, and adult) of *D. nuttalli* (Figure 2). The length of the PCR product was 224 bp, which is consistent with the expected size (Figure 2).

### Quantification of *Brucella* Genus-Specific Genes by TaqMan Real-Time PCR

**Figure 3:**
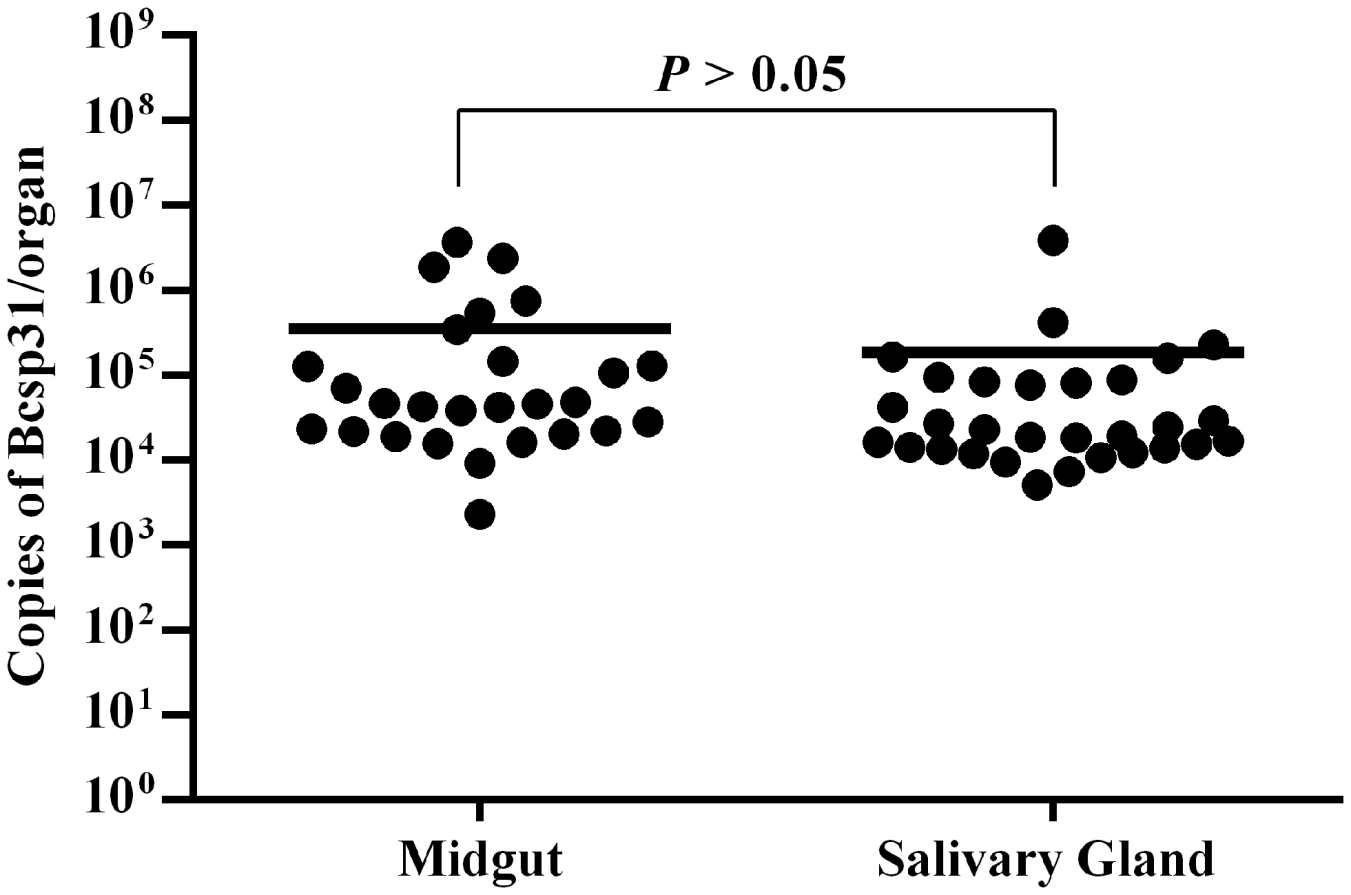
Copies of *Brucella* genus-specific genes from the midgut and salivary glands. Each data point represents a pool of five adult salivary glands or guts. The Y axis represents the total Bcsp31 gene copy number content of Brucella in the midgut and salivary glands. Non-parametric Mann–Whitney test showed significant differences in mean values (*P* < 0.05).

The salivary gland and midgut DNA samples were analyzed using the previously described methods. The average number of *Brucella* genus-specific gene copies ranged from 10^5^ to 10^6^ in different parts. Among them, the number of copies obtained from the midgut were higher than those obtained from the salivary gland (*P* > 0.05) (Figure 3).

### Bscp31 Protein of *Brucella* Detected in *D. nuttalli*

#### Bscp31 Protein of *Brucella* Detected in *D. nuttalli* by Immunofluorescence

**Figure 4:**
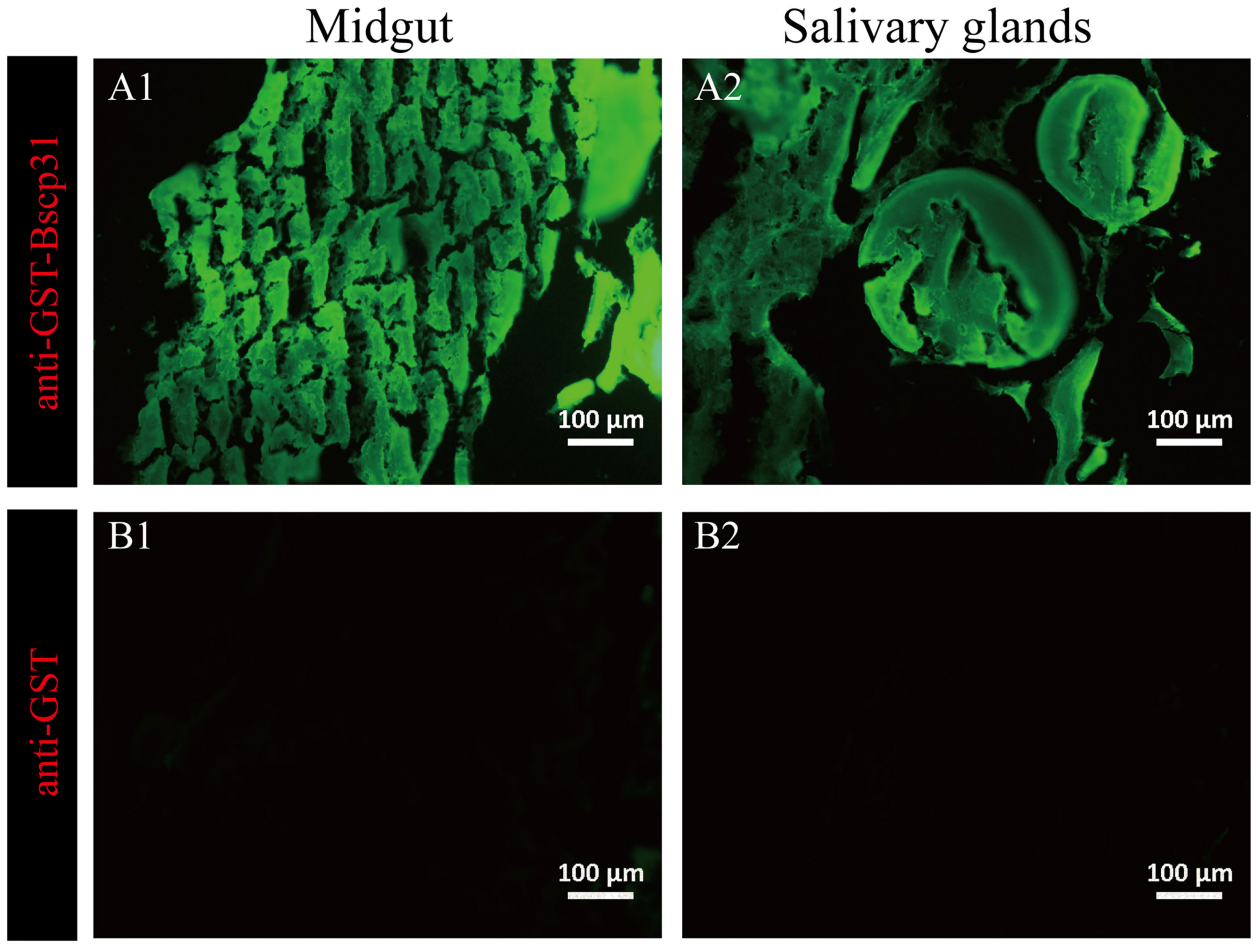
Immunofluorescence-based detection of Bscp31 protein in tick tissue samples. Representative images showing immunofluorescence-based detection of Bscp31 protein in tick tissue samples. Tissue sections were stained with goat anti-mouse antibodies (green, Alexa Green 488). Scale bars = 100 μm. Salivary glands **(A1,B1)** and midguts **(A2,B2)** of ticks. **(A1)** Salivary gland sections stained with anti-GST-Bscp31 antibodies. **(B1)** Salivary gland sections stained with anti-GST antibodies. **(A2)** Midgut sections stained with anti-GST-Bscp31 antibodies. **(B2)** Midgut sections stained with anti-GST antibodies.

The salivary glands incubated with mouse anti-GST-Bscp31 serum showed green fluorescence (Figure 4A1). Green fluorescence was also detected in the midgut incubated with mouse anti-GST-Bscp31 serum (Figure 4A2). Additionally, no fluorescence signal was detected in the salivary glands incubated with mouse anti-GST serum (Figure 4B1), and no fluorescence signal was detected in the midgut incubated with mouse anti-GST serum (Figure 4B2). These results indicate that the Bscp31 protein of *Brucella* is specifically present in the midgut and salivary glands of *D. nuttalli*.

#### Bscp31 Protein of *Brucella* Detected in *D. nuttalli* by Western Blotting

**Figure 5:**
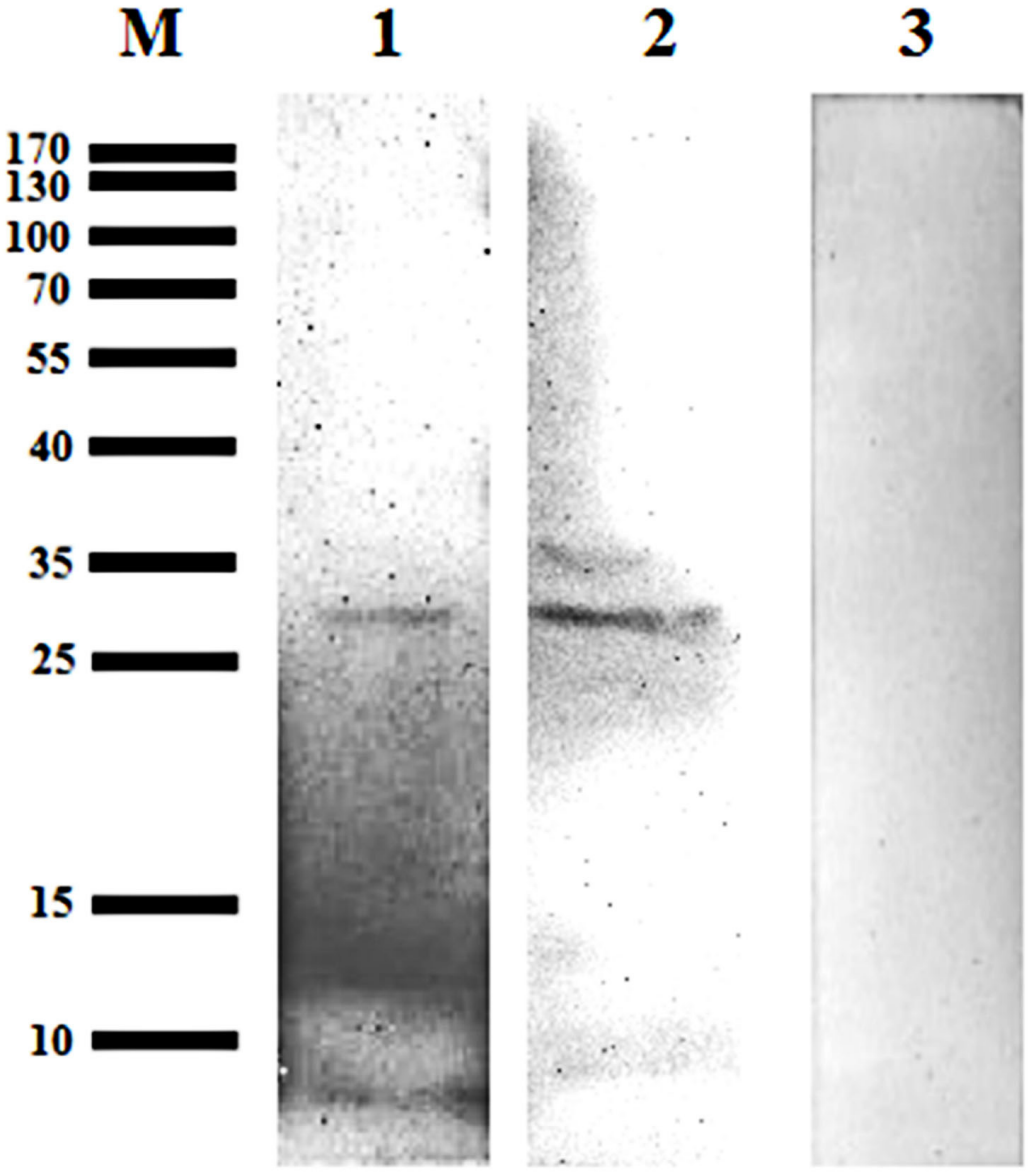
Detection of Bscp31 protein in *D. nuttalli* using Western blotting. Representative Western blot images. Lane M, prestained protein marker. Lane 1, Proteins that were extracted from midguts of female ticks and incubated with polyclonal antibodies from mice immunized with the GST-Bscp31 protein. Lane 2, Proteins that were extracted from salivary glands of female ticks and incubated with polyclonal antibodies from mice immunized with the GST-Bscp31 protein. Lane 3, Proteins that were extracted from female ticks and incubated with polyclonal antibodies from mice immunized with the GST protein.

A 31 kDa protein band was detected in the lysates from the midgut and salivary glands, when probed using mouse anti-GST-Bscp31 serum; the mouse anti-GST serum did not react with the midgut and salivary gland proteins (Figure 5). These results also indicate that the Bscp31 protein of *Brucella* is specifically located in the midgut and salivary glands of *D. nuttalli*.

### *Brucella* spp. Were Isolated From *D. nuttalli* and Identified

**Figure 6:**
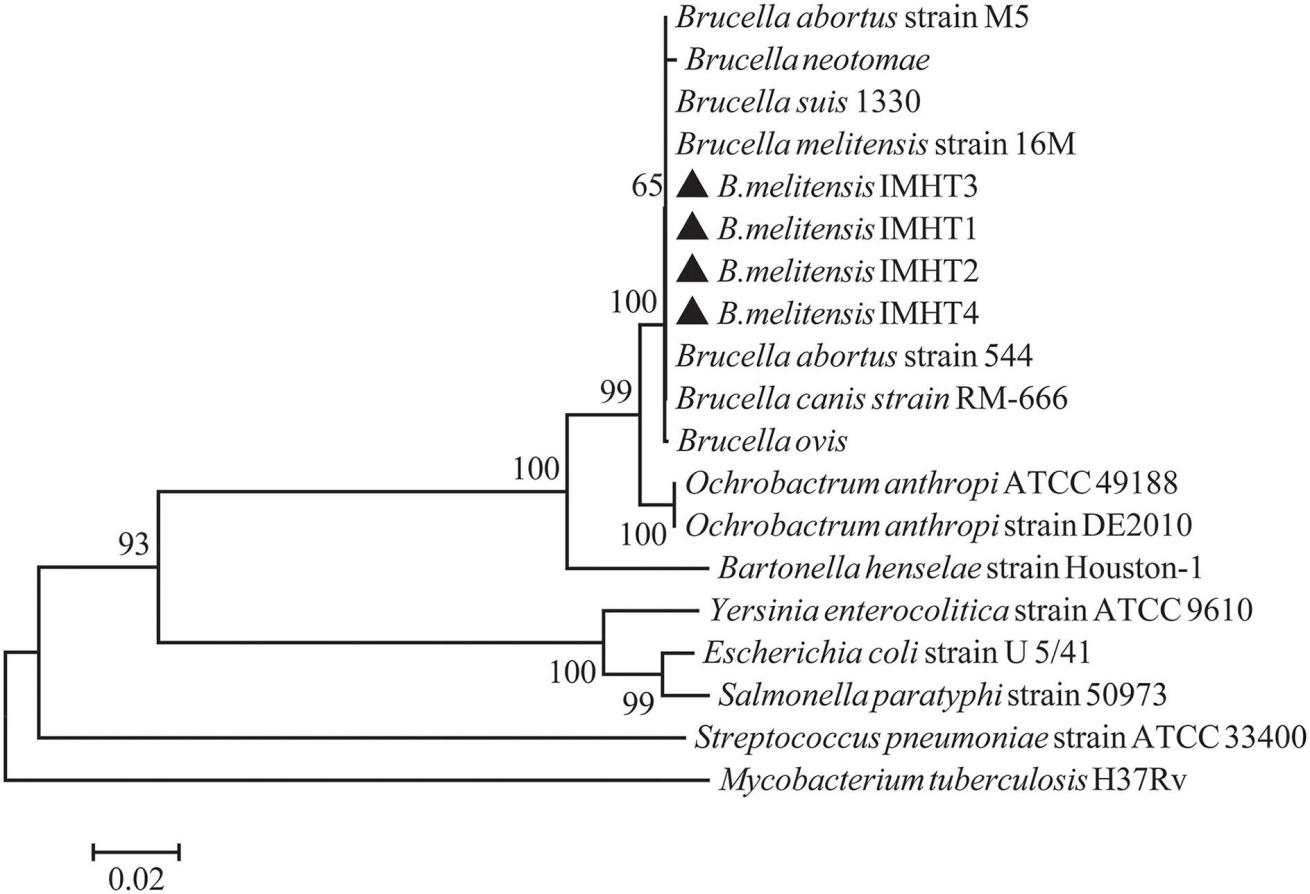
Phylogenetic relationships of *Brucella* spp. based on 16S rRNA sequences. Phylogenetic comparison of 16S rRNA sequences of *Brucella* spp. identified in this study (inverse color) and other sequences obtained from GenBank. Clustal W and the phylogenetic tree built using MEGA version 6 based on the neighbor-joining method. Values of 1,000 replications have been included.

**Figure 7:**
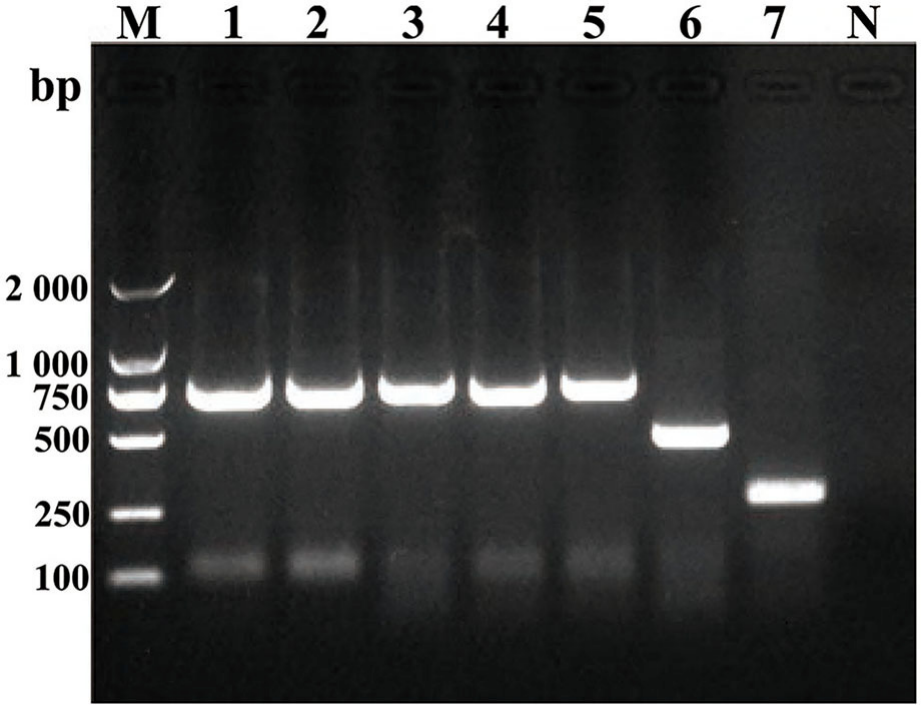
Amplification of DNA using AMOS-PCR assay. Amplification of DNA fragments from *Brucella* strains. DNA was amplified using the abbreviated AMOS PCR assay described in the text. Eight microliters of amplified product was separated by electrophoresis, treated with ethidium bromide, and visualized under UV light. Lane M, 2000 bp DNA marker. Lane 1, *B. melitenisi* NMT1. Lane 2, *B. melitenisi* NMT2. Lane 3, *B. melitenisi* NMT3. Lane 4, *B. melitenisi* NMT4. Lane 5, *B. melitenisi* strain 16M. Lane 6, *B. abortus* strain 544A. Lane 7, *B. suis* strain 1330S. Lane N, Negative control.

Some smooth, small, round, and dew-drop-like colonies appeared after 72 h of culture on *Brucella* selective medium. The phylogenetic tree showed that the test strains did not form branches with standard reference strains such as *B. melitensis, B. abortus, B. suis, B. canis, B. ovis*, and *B. neotomae*, but clustered closely on the same end branch, which did not clearly indicate the correlation between the strains. The isolated *Brucella* strains were named *B. melitensis* NMT1, *B. melitensis* NMT2, *B. melitensis* NMT3, and *B. melitensis* NMT4 (Figure 6). All the four strains identified, belong to *B. melitensis* subtype as confirmed by AMOS-PCR (Figure 7). Hence, we cultured *B. melitensis* alone and further classified it as *B. melitensis* biotype 3 according to phenotypic identification (Table 1).

**Table 1:** Characteristics of *Brucella* species according to classical biotyping.

**Experiments and results**		***B.melitenisi* NMT1**	***B.melitenisi* NMT2**	***B.melitenisi* NMT3**	***B.melitenisi* NMT4**	**International reference strains**		
		**Eggs of *D.nuttalli***	**Eggs of *D.nuttalli***	**Engorged *D.nuttalli***	**Engorged *D.nuttalli***	**16 M^**d**^**	**1330S^**d**^**	**544A^**d**^**
Dye inhibition^a^	Thionin	+	+	+	+	+	+	+
	Basic fuchsin	+	+	+	+	+	+	+
Lysis by phage at RTD^b^	Tb RTD 10^4^ Tb RTD BK2	− − +	− − +	− − +	− − +	− − +	− + +	+ + +
Agglutination on reaction with specific serum^c^	A M R	+ + −	+ + −	+ + −	+ + −	− + −	+ − −	+ − −
CO_2_ requirement		−	−	−	−	−	−	−
H_2_S production		−	−	−	−	+	+	+
Results	Species biotype	*Brucellamelitensis*3	*Brucella melitensis*3	*Brucellamelitensis*3	*Brucella melitensis*3	*Brucellamelitensis*1	*Brucella suis*1	*Brucellaabortus*1

a *Dye concentration of Brucella agar medium was 20 μg/mL*.

b *RTD: rate of Rational test dilution (the highest dilution rate at which the Tb phage completely lyses Brucella spp.)*.

c *A = A mono-specific serum; M = M mono-specific serum; R = R mono-specific serum*.

d *Reference strains were obtained from Chinese Center for Disease Control and Prevention*.

## Discussion

Brucellosis is a relatively old category of zoonotic diseases, with a wide range of vectors. It was once distributed worldwide; however, the use of attenuated vaccines in China, in as early as the 1980s, controlled its spread and reduced its incidence altogether (2). Unfortunately, reports on the re-emergence of this disease in multitude of animals have recently been published. Brucellosis has been reported in domestic animals, such as cattle, sheep, and pigs, as well as few wild animals (25). More interestingly, *Brucella* spp. have recently been isolated from amphibians (frogs) for the first time (26). Reports on widespread incidence of brucellosis infection among specific groups of individuals are of high concern (such as clinical veterinarians and herdsmen). This increase in the incidence of brucellosis can be attributed to a number of factors including deterioration in the quality of the vaccines, decreased stringency in the adapted animal feeding and management methods, variability of pathogens, changes in grassland macroclimate, and abnormal activity of arthropod vectors (8).

*Dermacentor nuttalli* is an arthropod, belonging to the order Ixodida of class Arachnida. It is a widely dominant tick in the Hulun Buir regions of Inner Mongolia. The peak activity period of *D. nuttalli* is between the months of April and June, every year. *Dermacentor nuttalli* is mainly an external parasite that sucks blood from various domestic animals. It causes direct injury to suck blood, which can also cause inflammation and skin allergy, often leading to pruritus in livestock. In addition, it can transmit a variety of infectious diseases (27–29). During the peak activity seasons of 2015 to 2019, 2,256 ticks were collected using different capturing methods in each site of the project area (Figure 1). PCR and real-time PCR methods were used to detect the *Brucella* genus-specific gene (*Bscp31* gene). We detected the presence of the *Brucella* spp. in the tick samples. To the best of our knowledge, this is the first report on the presence of this parasite in *D. nuttalli*. The detection rate is as high as 91% in female ticks and up to 56% in male ticks (detection data corresponding to samples collected from 2015 to 2016; Supplementary Table 1). This difference in the detection rates between the two sexes could be due to two possible reasons: (1) Female ticks have a longer life cycle and also exhibit more frequent and long-lasting blood-sucking behaviors than do male ticks. Consequently, the probability of carrying *Brucella* spp. is higher among female ticks than among male ticks. (2) Differences in sampling methods can also have an impact. We entrusted the livestock owners with the task of sample collection during 2015 to 2016. Collection of many tick samples from the same vector can be the reason behind such an excessively high detection rate among female ticks. Hence, this situation cannot be ruled out. Although these data are accurate for carrier rates of ticks, such a high detection rate should be confirmed further. Therefore, we adopted a different method of tick sample collection. Between 2017 and 2019, we visited the wild pastoral areas to ensure that no more than five blood-sucking ticks were collected per host, and also collected samples of nymphal ticks from the meadow. The consequent detection rate is as high as 66.67% in female ticks and up to 43.48% in the male ticks (detection data corresponding to samples collected from 2017 to 2019). Unexpectedly, because of the unique climate of the grassland, we failed to collect sufficient tick samples in 2018; thus, insufficient data were obtained for analysis for that year.

The 16S rRNA gene is one of the bacterial conserved genes; it has recently been used widely in research fields such as bacterial type identification and genetic evolution analysis. However, for the identification of *Brucella* spp., the 16S rRNA gene may not be very suitable. Reportedly, the gene shows a broad cross-reactivity with that of *Ochrobactrum anthropi* (30, 31). The current study has shown that *Bscp31* gene could be a key gene for the detection of *Brucella* spp.

To determine if *Brucella* can be carried by *D. nuttalli* for a long time, samples from ticks at different stages of development (such as the egg, larvae, nymph and adults) were evaluated. The results suggest that transovarian transmission of *Brucella* spp. occurs in *D. nuttalli*, thus confirming that ticks of this species can carry *Brucella* spp. for a long time (Figure 2). Previous studies on tick borne diseases transmitted by ticks reported that various pathogens are enriched in the salivary glands and midguts of ticks. This finding indicates that pathogens are lodged in vectors and are transmitted when these vectors bite other healthy animals (32). In this study, both gene and the protein level analyses showed evidence of *Brucella* infection in the midgut and salivary glands of *D. nuttalli* (Figures 3–5). *Brucella* spp. are indeed effectively enriched in the two key tissues i.e., the midgut and salivary glands of *D. nuttalli*. The above-mentioned results suggest that the risk of arthropod-bite mediated transmission of brucellosis to other animals or to humans is high.

In addition, we have successfully isolated four strains of *Brucella* from *D. nuttalli*. The *Bscp31* gene and 16S rRNA gene of *Brucella* were selected for amplification, and the gene sequences of 16S rRNA in all the four strains were obtained. Moreover, the phylogenetic tree of the 16S rRNA gene of *Brucella* was mapped using Mega 6 (Figure 6). The phylogenetic tree showed that the test strains did not form branches with standard reference strains such as *B. melitensis, B. abortus, B. suis, B. canis, B. ovis*, and *B. neotomae*, but gathered closely at the same terminal branch, thus indicating that the isolated strains were those of *Brucella*. However, 16S rRNA of *Brucella* is highly conserved, and the sequence similarity between strains is very high; therefore, the correlation between individual strains cannot be clearly identified. In successive studies, AMOS-PCR method and typing identification test confirmed that the isolated strains belong to *B. melitensis* biotype 3 and were named as *B. melitensis* NMT1, *B. melitensis* NMT2, *B. melitensis* NMT3, and *B. melitensis* NMT4, respectively (Figure 7 and Table 1).

According to the molecular epidemiological survey, in the past 5 years, the areas where brucellosis epidemic were observed are also areas where tick activity is rampant; therefore, ticks are likely to be novel arthropod hosts for brucellosis. However, the question of whether or not ticks can transmit brucellosis to other animals or humans remains to be addressed further in laboratories with higher biosafety standards.

## Conclusion

This study confirmed that *D. nuttalli* can carry *Brucella* spp. for a long time and that the former might show transovarial transmission of the latter, which highlights the possibility of long-term propagation of brucellosis. Our studies also reveal the abundance of *Brucella* spp. in the midgut and salivary glands of *D. nuttalli*. Furthermore, we have successfully isolated four strains of *Brucella* from *D. nuttalli*. In conclusion, we can suggest that bites from *D. nuttalli* are associated with the potential risk of transmission of brucellosis to animals and even humans.

## Data Availability Statement

The 16S rRNA genes of Brucella IMHT1 to IMHT4 strains isolated have been submitted to Genbank, and the corresponding Accession Numbers were MT611102, MT611103, MT611104, and MT611105.

## Author Contributions

G made substantial contributions to conception, design and coordination of the study, drafted the manuscript and gave final approval of the version to be published. JW provides a Biosafety Level-3 laboratory environment for *Brucella* isolation and identification test. TH carried out the experimental part and data analysis, and completed the final manuscript. JZ collected ticks, performed the pooled sampling. CS assisted in the isolation and identification of *Brucella*. ZL and HH provided assistance to this work. All authors read and approved the final manuscript.

## Conflict of Interest

The authors declare that the research was conducted in the absence of any commercial or financial relationships that could be construed as a potential conflict of interest.
